# Potential Survival and Pathogenesis of a Novel Strain, *Vibrio parahaemolyticus* FORC_022, Isolated From a Soy Sauce Marinated Crab by Genome and Transcriptome Analyses

**DOI:** 10.3389/fmicb.2018.01504

**Published:** 2018-07-06

**Authors:** Han Y. Chung, Byungho Lee, Eun J. Na, Kyu-Ho Lee, Sangryeol Ryu, Hyunjin Yoon, Ju-Hoon Lee, Hyeun B. Kim, Heebal Kim, Hee G. Jeong, Bong-Soo Kim, Sang H. Choi

**Affiliations:** ^1^Department of Agricultural Biotechnology, Center for Food Safety and Toxicology, Seoul National University, Seoul, South Korea; ^2^Food-borne Pathogen Omics Research Center (FORC), Seoul National University, Seoul, South Korea; ^3^Department of Life Science, Sogang University, Seoul, South Korea; ^4^Department of Applied Chemistry & Biological Engineering, Ajou University, Suwon, South Korea; ^5^Department of Food Science and Biotechnology, Kyung Hee University, Yongin, South Korea; ^6^Department of Animal Resources Science, Dankook University, Cheonan, South Korea; ^7^Department of Animal Science and Biotechnology, Seoul National University, Seoul, South Korea; ^8^Department of Food Science and Technology, Chungnam National University, Daejeon, South Korea; ^9^Department of Life Science, Multidisciplinary Genome Institute, Hallym University, Chuncheon, South Korea

**Keywords:** *Vibrio parahaemolyticus*, whole genome sequencing, genomic comparison, transcriptome, virulence factors, crab, FORC_022

## Abstract

*Vibrio parahaemolyticus* can cause gastrointestinal illness through consumption of seafood. Despite frequent food-borne outbreaks of *V. parahaemolyticus*, only 19 strains have subjected to complete whole-genome analysis. In this study, a novel strain of *V. parahaemolyticus*, designated FORC_022 (Food-borne pathogen Omics Research Center_022), was isolated from soy sauce marinated crabs, and its genome and transcriptome were analyzed to elucidate the pathogenic mechanisms. FORC_022 did not include major virulence factors of thermostable direct hemolysin (*tdh*) and TDH-related hemolysin (*trh*). However, FORC_022 showed high cytotoxicity and had several *V. parahaemolyticus* islands (VPaIs) and other virulence factors, such as various secretion systems (types I, II, III, IV, and VI), in comparative genome analysis with CDC_K4557 (the most similar strain) and RIMD2210633 (genome island marker strain). FORC_022 harbored additional virulence genes, including accessory cholera enterotoxin, zona occludens toxin, and tight adhesion (tad) locus, compared with CDC_K4557. In addition, O3 serotype specific gene and the marker gene of pandemic O3:K6 serotype (*toxRS*) were detected in FORC_022. The expressions levels of genes involved in adherence and carbohydrate transporter were high, whereas those of genes involved in motility, arginine biosynthesis, and proline metabolism were low after exposure to crabs. Moreover, the virulence factors of the type III secretion system, tad locus, and thermolabile hemolysin were overexpressed. Therefore, the risk of foodborne-illness may be high following consumption of FORC_022 contaminated crab. These results provided molecular information regarding the survival and pathogenesis of *V. parahaemolyticus* FORC_022 strain in contaminated crab and may have applications in food safety.

## Introduction

*Vibrio parahaemolyticus* is curved rod-shape, Gram-negative halophilic bacterium with a single polar flagellum and is abundant in estuarine environments and various seafood, including shellfish, oysters, clams, cockles, crabs, and shrimps (Joseph et al., 1982; Newton et al., 2014; Rodgers et al., 2014; Xu et al., 2014; Malcom et al., 2015). This bacterium causes seafood borne gastroenteritis, and its infection is usually associated with the consumption of raw or undercooked seafood (Daniels et al., 2000; Letchumanan et al., 2016). Recently, the occurrence of food borne disease and outbreaks due to seafood contamination with *V. parahaemolyticus* have increased significantly around the world (Letchumanan et al., 2015a,b). The symptoms include watery diarrhea, vomiting, nausea, abdominal cramps, septicemia and even death (Hazen et al., 2015). To understand the pathogenicity of *V. parahaemolyticus*, several studies have analyzed the association of its virulence factors with food poisoning (Haendiges et al., 2015; Li et al., 2017). The complete genomes of 19 strains of *V. parahaemolyticus* have been sequenced and are currently available from the National Center for Biotechnology Information (NCBI) database^1^.

*Vibrio parahaemolyticus* strains isolated from patients hospitalized due to food-borne illness harbor multiple virulence genes, including thermostable direct hemolysin (*tdh*) and TDH-related hemolysin (*trh*) (Kishishita et al., 1992). These genes are indicators of *V. parahaemolyticus* pathogenicity and elicit enterotoxic effects on human intestinal cells (Ottaviani et al., 2012; Ludeke et al., 2015). For example, *V. parahaemolyticus* KCTC 2471 (=ATCC 33844 = CDC strain KC 824), which harbored *tdh* gene as major virulence factor, caused food poisoning in Japan (Baumann et al., 1971; Hossain et al., 2013). However, several strains isolated from clinical samples were found to be negative for *tdh* and *trh* genes, suggesting that these strains may carry some other virulence factors (Park et al., 2004a; Lynch et al., 2005; Velazquez-Roman et al., 2012). Genes related to type III secretion, tight adhesion locus (tad locus), and hemolysin have been suggested as possible contributors to *V. parahaemolyticus* pathogenicity (Tomich et al., 2007; Caburlotto et al., 2010). Various secretion systems (types I, II, III, IV, and VI) are conserved in Gram-negative bacteria and are known to play a role in pathogenicity of *V. parahaemolyticus* by mediating the transportation of virulence-related proteins across the bacterial membrane (Pallen et al., 2003; Delepelaire, 2004; Park et al., 2004b; Abendroth et al., 2009; Wang et al., 2015). In addition, *V. parahaemolyticus* islands (VPaI), which are located in pathogenic islands in *V. parahaemolyticus*, are thought to play a role in the pathogenicity of this organism (Hurley et al., 2006). Infection of human cell lines by *V. parahaemolyticus* lacking *tdh* and *trh* genes also results in significant cytotoxicity (Broberg et al., 2011; Ritchie et al., 2012). Therefore, major virulence factors of *V. parahaemolyticus* have still not been identified at the genomic level.

About 28% of outbreaks by *V. parahaemolyticus* attributed to a single food commodity are due to aquatic products, such as crustaceans and shellfish (Wu et al., 2014). Crabs are a major source of *V. parahaemolyticus* outbreaks among aquatic products in northeast Asian due to improper cooking and wound infection from mishandling (Rodgers et al., 2014). However, no reports have described whole-genome sequences of *V. parahaemolyticus* isolated from contaminated crab; therefore, no information is available regarding the major virulence factors of *V. parahaemolyticus* obtained from crabs. Furthermore, information regarding the expression of virulence factors of *V. parahaemolyticus* in contaminated food is limited. Because crabs are a popular source of seafood in Asia, analyses of whole-genome sequences and the transcriptome of *V. parahaemolyticus* isolated from crab products are necessary to investigate the potential risks of foodborne illnesses from contaminated products.

In this study, we performed complete whole-genome sequencing of *V. parahaemolyticus* FORC_022 strain, isolated from soy sauce marinated swimming crabs in South Korea, and compared with complete sequences of this species from the public database. In addition, the transcriptome of this strain was analyzed to elucidate the potential pathogenicity of this strain in crabs using an artificial contact experiment. These results provide a better understanding of the pathogenicity of *V. parahaemolyticus* in crab-based foods and could facilitate the prevention of foodborne illness.

## Materials and Methods

### Isolation, Growth Conditions and Morphology

FORC_022 strain was isolated from soy sauce marinated crabs by the Jeollanam-do Institute of Health and Environment, South Korea. The strain was cultivated aerobically at 30°C in modified Luria-Bertani medium supplemented with 1% (w/v) NaCl for 12 h (Sakazaki, 1983).

The cells were negatively stained with uranyl acetate for 1 min and were then observed using a transmission electron microscopy (JEM-2100; JEOL, Tokyo, Japan) at 200 kV. The FORC_022 strain was a curved, rod-shaped bacterium that was 1.5–2.0 μm in length and 0.6–0.8 μm in width with a single polar flagellum (Supplementary Figure S1).

PCR amplification was performed to determine serotype of FORC_022 strain using genetic markers of O-serotypes specific genes and *toxRS* sequences unique to the pandemic O3:K6 serotype of *V. parahaemolyticus* (Supplementary Figure S2) (Matsumoto et al., 2000; Nasu et al., 2000; Chen et al., 2012; Akther et al., 2016).

### Cytotoxicity Test

Cytotoxicity of the FORC_022 strain was evaluated by measuring the activity of cytoplasmic lactate dehydrogenase (LDH) released from INT-407 human epithelial cells (ATCC, Manassas, VA, United States) after the plasma membrane was damaged. INT-407 cells were grown in minimum essential medium containing 1% (v/v) fetal bovine serum (MEMF; Gibco-BRL, Gaithersburg, MD, United States) in 96-well culture dishes (Nunc, Roskilde, Denmark) as described previously (Kim et al., 2014). Cells (2 × 10^4^ cells/well) were infected with FORC_022 or KCTC 2471 (control) at various multiplicities of infection (MOI) for 4 h. The MOI is the ratio for the number of bacterial cells to the number of epithelial cells. *V. parahaemolyticus* KCTC 2471 strain, which was isolated from patients of food poisoning in Japan, was used as control in LDH assay (Baumann et al., 1971). The LDH activity in the supernatants was determined using a cytotoxicity detection kit (Roche, Mannheim, Germany).

### Genome Sequencing and Annotation

Genomic DNA was extracted from cultured strains using a DNeasy Blood & Tissue Kit (Qiagen, Valencia, CA, United States) according to the manufacturer’s protocol. Contamination of pure cultured strain was verified using 16S rRNA sequencing, and taxonomic identification was conducted by phylogenetic tree analysis using MEGA6 (Tamura et al., 2013). The phylogenetic tree was constructed using the neighbor-joining method with 1,000 bootstrap replicates. Genome sequencing was conducted at ChunLab, Inc. (Seoul, South Korea) using hybrid sequencing of Illumina MiSeq (Illumina, San Diego, CA, United States) and a PacBio RS II system (Pacific Biosciences, Menlo Park, CA, United States) according to the manufacturers’ protocols. Raw sequences obtained from PacBio RS II were assembled by PacBio SMRT Analysis ver. 2.3.0 software (Pacific Biosciences), and raw sequences from Illumina MiSeq were assembled by CLC Genomics Workbench ver. 7.5.1 (CLC bio, Aarhus, Denmark). The hybrid assembly of generated contigs from both systems was performed using the CodonCode Aligner (CodonCode, Co., Dedham, MA, United States).

Open reading frames (ORFs) and annotations were predicted by the Rapid Annotation using Subsystem Technology (RAST) server (Aziz et al., 2008) and GeneMarkS program (Besemer et al., 2001). The ribosome binding sites were predicted using RBSfinder (J. Craig Venter Institute, Rockville, MD, United States). Subsequent predictions of the functions of ORFs and their conserved protein domains were carried out using InterProScan 5 (Jones et al., 2014) and COG-based WebMGA programs (Wu et al., 2011). The circular genome maps were drawn using the GenVision program (DNASTAR, Madison, WI, United States). The putative virulence factors of FORC_022 were characterized using BLAST against the Virulence Factor Database^2^ (Chen et al., 2005).

### Comparative Genome Analysis

The genome tree was used to determine the closest strain to FORC_022 among completely sequenced *V. parahaemolyticus* strains (CDC_K4557, ATCC 17802, BB22OP, FDAARGOS_191, RIMD 2210633, 10329, FORC_008, FORC_018, FORC_014, FORC_006, MAVPQ, MAVP-Q, UCM-V493, FORC_023, CHN25, MAVP-R, FORC_004, and FDA_R31) based on ANI values (Supplementary Figure S3). The ANI values were obtained from whole-genome sequences of strains using the JSpecies program (Richter and Rossello-Mora, 2009) by comparing sequences fragmented into 1,020-bp sections based on BLAST analysis. The genome tree was constructed using the R program (3.4.4). A comparative genome analysis between FORC_022 and CDC_K4557 was conducted with the Artemis Comparison Tool (ACT) (Carver et al., 2005). Pangenome analysis of FORC_022 with CDC_K4557 and RIMD 2210633 was conducted using GView Server^3^.

### Artificial Contact With Swimming Crab and RNA Extraction From the FORC_022 Strain

To identify the potential survival mechanism and pathogenicity of FORC_022 contaminating swimming crab during handling or cooking, we artificially exposed crab to the FORC_022 strain. FORC_022 was grown to mid-log phase (*A*_600_ of 0.8) in *V. fischeri* minimal medium containing glycerol and then exposed to crab for 4 h. The *V. fischeri* minimal medium containing glycerol was used for artificial seawater medium for *Vibrio* (Cao et al., 2012; Kim et al., 2013). FORC_022 strain without exposure to crabs in the same medium was also prepared as a negative control. These experiments were performed in triplicate. The culture was filtrated with a syringe, sterilized gauze and a vacuum filter with Whatman no.1 filter paper (Whatman International Ltd., Maidstone, England). Subsequently, the filtered product was transferred to 50 mL falcon tubes (SPL, Kyungki, South Korea) and centrifuged at 5,000 × *g* and 4°C for 10 min. The pellets were resuspended in 0.5 mL cold diethyl phosphorocyanidated-treated phosphate-buffered saline after centrifugation, and the solutions were then mixed with 1 mL RNAprotect Bacteria Reagent (Qiagen). Total RNAs were isolated using a miRNeasy Mini Kit (Qiagen) according to the manufacturer’s protocol. DNA contaminations were removed using TURBO DNase (AMbion, Austin, TX, United States), and extracted RNAs were then cleaned up using an RNeasy MinElute Cleanup kit (Qiagen). The extracted RNA was verified with an Agilent 2100 Bioanalyzer with Agilent RNA 6000 Nano reagents (Supplementary Table S1) (Agilent Technologies, Waldbronn, Germany).

### Transcriptome Analysis

To sequence the RNA, mRNA was enriched from extracted total RNA by depleting rRNAs by using a Ribo-Zero^TM^ rRNA Removal kit (Epicentre, Madison, WI, United States). The cDNA library was constructed from enriched mRNA using a TruSeq Stranded mRNA Sample Preparation kit (Illumina) following the manufacturer’s instructions. The quality of cDNA libraries was determined using an Agilent 2100 Bioanalyzer (Agilent Technologies). Strand-specific paired-ended 100-nucleotide reads from each cDNA library were obtained using HiSeq2500 (Illumina). For biological replication, two libraries were separately constructed and sequenced from RNAs isolated from two independently filtered culture supernatants of FORC_022.

Reads obtained from RNA sequencing were mapped to the FORC_022 reference genome using CLC Genomics Workbench ver. 7.5.1 (CLC Bio). The relative transcript abundance was measured by reads per kilobase of transcript per million mapped sequence reads (Mortazavi et al., 2008). Genes with two or greater fold change with *p*-values < 0.01 were considered differentially expressed in samples using the CLRNASeq^TM^ program (ChunLab). Transcriptome analysis and visualization of virulence gene data were carried out using Gitools (Perez-Llamas and Lopez-Bigas, 2011).

### Quantitative Real Time PCR (qRT-PCR)

Extracted RNA was converted to cDNA for qRT-PCR using an iScript cDNA Synthesis Kit (Bio-Rad, Hercules, CA, United States). Real-time PCR of cDNA was performed using a Chromo 4 Real-time PCR Detection system (Bio-Rad) with SYBR Green I (Kim et al., 2013). The primers used in this study are listed in Supplementary Table S2. Relative expression levels of specific transcripts were calculated using the 16S rRNA and *recA* expression level as an internal reference for normalization (Ma et al., 2015). The amplification efficiencies and stability of reference genes were calculated using Delta CT method in Bio-Rad CFX Manager software ver. 3.1 (Supplementary Table S3). qRT-PCR data are presented as mean ± standard deviation of three independent experiments. The differences between groups were determined using two-tailed *t*-tests in SigmaPlot software (ver. 12.0).

### Sequence Deposit in Public Databases

The whole genome sequence of *V. parahaemolyticus* FORC_022 was deposited in the GenBank of NCBI^4^ under accession numbers CP013248, CP013249 and CP013250 for chromosomes I, II, and a plasmid, respectively. Transcriptome analysis data of virulence factors in *V. parahaemolyticus* FORC_022 was deposited in the NCBI Sequence Read Archive (SRA) under the accession number SRP135619.

## Results and Discussion

### General Genome Features

The FORC_022 strain was isolated from a soy sauce marinated crab and identified as a species of *V. parahaemolyticus* by phylogenetic tree analysis of the 16S rRNA gene (Supplementary Figure S4). The genome of FORC_022 consisted of two circular chromosomes and a plasmid with 5,379,414 bp with 45.25% GC content (Supplementary Table S4). Chromosome I consisted of 3,397,828 bp with 45.20% GC content and contained 3,066 predicted ORFs, 119 tRNA genes, and 34 rRNA genes. Moreover, 2,423 ORFs (79.02%) were predicted to be functional, and 643 ORFs (17.71%) were predicted to encode hypothetical proteins. Chromosome II consisted of 1,879,989 bp with 45.37% GC content and contained 1,685 predicted ORFs, 14 tRNA genes, and three rRNA genes. Among the ORFs, 1,322 (78.46%) were predicted to be functional, and 363 (21.54%) were predicted to encode hypothetical proteins. The plasmid pFORC22 consisted of 100,597 bp with 44.49% GC content containing 107 predicted ORFs. Of these, 41 ORFs (38.32%) were predicted to be functional, and 66 ORFs (61.68%) were predicted to encode hypothetical proteins. Genome maps of chromosomes and a plasmid are shown in Supplementary Figure S5.

### Pathogenesis and Virulence Factors

**FIGURE 1:**
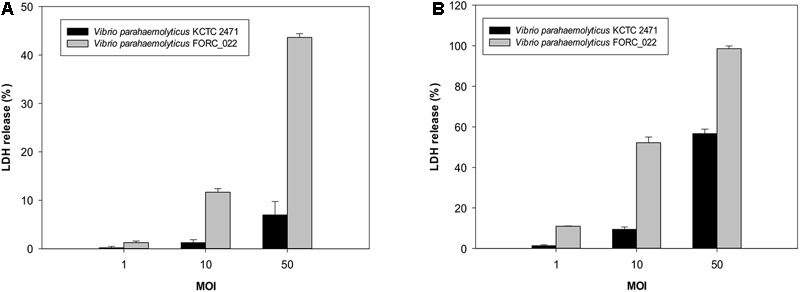
Cytotoxicity analyses of the FORC_022 strain were compared with the KCTC 2472 strain by measuring the activity of cytoplasmic lactate dehydrogenase (LDH). INT-407 cells were infected with FORC_022 or KCTC 2471 at various multiplicities of infection (MOIs) for **(A)** 2 h and **(B)** 3 h. Cytotoxicity was determined as the percentage of LDH leakage using the amount of LDH from the cells that were completely lysed by 2% Triton X-100. Error bars represent the standard errors of the means.

The cytotoxicity of FORC_022 was compared with KCTC 2471 (=ATCC 33844 = CDC strain KC 824) strain as a positive control, which was isolated from a food poisoning patient (Baumann et al., 1971; Kim et al., 2012; Hossain et al., 2013). The cytotoxicity of FORC_022 was higher than that of KCTC 2471 strain in lactate dehydrogenase (LDH) release assays (**Figure 1**). However, FORC_022 did not encode major virulence factors such as *tdh* and *trh genes*, which are correlates with the Kanagawa phenomenon and induce significant cytotoxicity (Kishishita et al., 1992; Raghunath, 2014). Most strains of *V. parahaemolyticus* usually contain *tdh* and *trh* genes, which have been shown to be associated with pathogenicity, leading to clinical outbreaks (DePaola et al., 2000, 2010; Broberg et al., 2011; Ludeke et al., 2015). The TDH and TRH toxins produced by *V. parahaemolyticus* can invade the host and disrupt its membrane leading to hemolysis and cytotoxicity (Casandra et al., 2013). However, recent studies reported that *V. parahaemolyticus* isolated from clinical sources induced cytotoxicity without *tdh* and/or *trh* genes (Broberg et al., 2011). In addition, mutant strains of Δ*tdh/*Δ*trh* show significant cytotoxicity, suggesting that other virulence factors of *V. parahaemolyticus* may be involved in its pathogenicity (Xu et al., 1994; Park et al., 2004b; Lynch et al., 2005; Pazhani et al., 2014). Consistent with this finding, our results showed that *V. parahaemolyticus* FORC_022 isolated from a soy sauce marinated crab caused cytotoxicity in human cell lines without the *tdh* and *trh* genes. This result indicated that the FORC_022 strain could contain other virulence factors. To identify virulence factors of FORC_022 related to cytotoxicity, the virulence factors were searched for known virulence factors in the Virulence Factor Database (Chen et al., 2005) and annotated information for the FORC_022 genome (Supplementary Table S5).

Several ORFs encoding various hemolysin genes (FORC22_0344, FORC22_0414, FORC22_1716, FORC22_2576, and FORC22_3059 in chromosome I; FORC22_3287, FORC22_3317, and FORC22_3346 in chromosome II) were detected; these genes may be responsible for the virulence of the FORC_022 strain. In addition, various secretion systems including types I, II, III, IV, and VI (T1SS, T2SS, T3SS, T4SS, and T6SS) were detected in the genome sequence of the FORC_022 strain. Type I secretion systems were detected from FORC22_0395, FORC22_1432, FORC22_1615-1620, and FORC22_2067 in chromosome I and FORC22_4335, and FORC22_4733-4736 in chromosome II; these secretion systems are required for the secretion of repeat-in-toxin (RTX; the major virulence factor of *V. cholerae*) (Boardman et al., 2007). Type II secretion systems were detected from FORC22_2454 to FORC22_2462 in chromosome I and from FORC22_3787 to FORC22_3862 in chromosome II; these secretion systems are required for secretion of the cholera toxin (the major virulence factor of *V. cholerae*) (Abendroth et al., 2009). Type III secretion systems, which serve several pathogenic functions, such as apoptosis and autophagy (Ono et al., 2006), were detected in a region of chromosome I (FORC22_1641-1687). T3SS has been shown to be involved in the cytotoxicity of *V. parahaemolyticus* in eukaryotic cells (Park et al., 2004b; Kodama et al., 2007). Moreover, T3SS2 effectors are translocated into the host cell membrane to cause enterotoxicity in colon epithelial cells. Type IV secretion systems, which are associated with effector protein injection machinery by disrupting the actin cytoskeleton or by inducing cell death pathways in host immune cells (Ham et al., 2011), were detected in the region from FORC22_2454 to FORC22_2462 in chromosome I and from FORC22_3787 to FORC22_3862 in chromosome II. Type VI secretion systems act as toxin proteins by delivering bacterial proteins into eukaryotic cells and causing cell death (Costa et al., 2015); these regions were detected from FORC22_1401 to FORC22_1409 in chromosome I and FORC22_4148 to FORC22_4166 in chromosome II. In addition, *V. parahaemolyticus* has two *icmF* family genes (*icmF1* and *icmF2*) in type VI secretion systems, which contribute to pathogenicity including adhesion to host epithelial cells and cytotoxicity (Yu et al., 2012). The *icmF1* was detected at FORC22_1405 and the *icmF2* was detected at 1,086,583–1,086,627 position in chromosome I. Therefore, the FORC_022 may have the potential to induce pathogenesis through these virulence factors. Further studies are necessary to verify the effects of these secretion systems on disease onset.

Iron uptake from host cells can play a key role in survival of *V. parahaemolyticus* leading to its pathogenicity. Previous study reported that iron loss caused by *V. parahaemolyticus* affects the integrity of heme protein and leads to host cell death (Hurley et al., 2006). FORC_022 included enterobactin receptors (detected at the FORC22_2639 region on chromosome I and FORC22_3685 region on chromosome II), heme receptors (FORC22_4014 and FORC22_4477 regions on chromosome II), and iron ABC transport (FORC22_3679 to FORC22_3682 on chromosome II) (Supplementary Table S5). These iron uptake-related genes may affect the survival of FORC_022 in host cells.

### Comparative Genome Analysis of FORC_022

**FIGURE 2:**
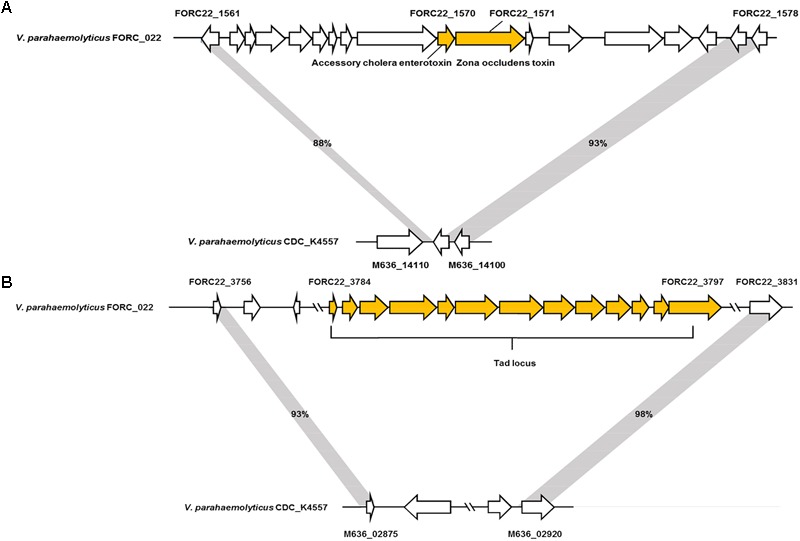
Two non-homologous regions between FORC_022 and CDC_K4557 strains. **(A)** Different regions detected in chromosome I and **(B)** chromosome II of FORC_022.

The genome tree of FORC_022 with other completely sequenced *V. parahaemolyticus* strains (CDC_K4557, ATCC 17802, BB22OP, FDAARGOS_191, RIMD 2210633, 10329, FORC_008, FORC_018, FORC_014, FORC_006, MAVPQ, MAVP-Q, UCM-V493, FORC_023, CHN25, MAVP-R, FORC_004, and FDA_R31) was obtained based on ANI values (Supplementary Figure S3), and the general features of genomes were compared (**Table 1**). The highest ANI values (98.54%) were detected for FORC_022 and CDC_K4557 strain (**Table 1**), the latter of which was isolated from the stool of a patient in Louisiana in 2007 and was submitted to the Centers for Disease Control and Prevention (CDC) (Morrison et al., 2012). Significantly different regions between FORC_022 and CDC_K4557 were detected ranging from positions 1,720,159 to 1,726,760 (FORC22_1562 to FORC22_1576) in chromosome I (**Figure 2A** and Supplementary Figure S6A). These regions were only detected in the FORC_022 strain and contained the accessory cholera enterotoxin (FORC22_1570) and zona occludens toxin (FORC22_1571), which are associate with colonization and pathogenesis (Tacket et al., 1993; Mukhopadhyay et al., 1995). Accessory cholera enterotoxin contributes to intestinal secretion and diarrhea by stimulating Ca^2+^-dependent Cl^-^/HCO^3-^ symporters (Trucksis et al., 2000), whereas zona occludens toxin weakens intestinal tight junctions, leading to body fluid secretion into the intestinal lumen (Fasano et al., 1997). Another different region was detected in chromosome II (ranging from FORC22_3757 to FORC22_3830; positions 750,924-830,901; **Figure 2B** and Supplementary Figure S6B). The tad locus was detected only in FORC_022 (ranging from FORC22_3784 to FORC22_3797) and has been shown to be related to biofilm formation, colonization, and pathogenicity of strains, thereby leading to several diseases in both humans and animals (Tomich et al., 2007; Morrison et al., 2012). Although the level of cytotoxicity in human cell lines cannot be directly compared between FORC_022 and CDC_K4557 due to unavailability of CDC_K4557, the presence of additional virulence factors may contribute to the cytotoxicity of FORC_022 in various human cells lines. CDC_K4557 did not harbor other virulence factors, such as the tad locus, which mediates the formation of biofilms and facilitates survival by utilizing nutrients from the host and protecting the organism from host immune surveillance detected in *Pasteurella multocida* and *Yersinia ruckeri* (Fuller et al., 2000; Fernandez et al., 2004). Although the role of the tad locus in pathogenicity has not yet been clarified, our data demonstrated the existence of the tad locus in *V. parahaemolyticus*. These results indicated that FORC_022 may be pathogenic to humans since its genome contains several putative virulence factors.

**Table 1:** Genome features of completely sequenced *Vibrio parahaemolyticus* strains and average nucleotide identity (ANI) values to FORC_022.

Strains	Total genome size (Mb)	Average G + C content (%)	Number of plasmids	Number of proteins	Number of tRNAs	Numbrt of rRNAs	ANI value to FORC_022 (%)	BioProject no.
**FORC_022**	5.34	45.26	1	4,844	133	37	–	PRJNA301198
CDC_K4557	5.14	45.34	0	4,461	130	34	98.54	PRJNA203445
ATCC_17802	5.16	45.33	0	4,652	134	43	98.52	PRJNA231221
BB22OP	5.11	45.33	0	4,642	156	34	98.52	PRJNA170885
FDAARGOS_191	5.18	45.4	0	4,624	132	37	98.52	PRJNA231221
RIMD 2210633	5.17	45.4	0	3,831	156	34	98.52	PRJNA360
10329	5.15	45.3	0	4,829	127	31	98.46	PRJNA231221
FORC_008	5.04	45.44	0	4,624	130	32	98.44	PRJNA266097
FORC_018	5.04	45.44	0	4,510	132	37	98.44	PRJNA303095
FORC_014	5.19	45.35	1	4,845	125	34	98.43	PRJNA280138
FORC_006	5.1	45.33	0	4,707	132	37	98.41	PRJNA261558
MAVPQ	5.26	45.3	0	4,840	135	41	98.41	PRJNA286197
MAVP-Q	5.26	45.3	0	4,841	135	41	98.41	PRJNA263814
UCM-V493	5.24	45.32	1	4,795	122	28	98.39	PRJNA229758
FORC_023	5.01	45.44	0	4,552	131	37	98.39	PRJNA284329
CHN25	5.44	45.19	3	4,781	107	28	98.39	PRJNA274308
MAVP-R	5.38	44.68	2	4,882	135	43	98.38	PRJNA263814
FORC_004	5.17	45.49	1	4,713	132	39	98.36	PRJNA259940
FDA_R31	5.22	45.33	0	4,563	130	37	98.26	PRJNA203445

**FIGURE 3:**
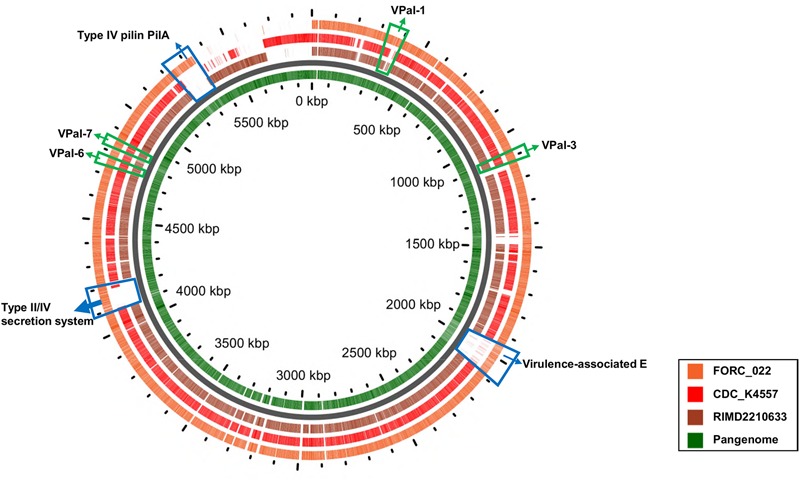
A pangenome analysis between three *V. parahaemolyticus* strains. The central circle belongs to the main bacterial strain, and the outer circles belong to other strains. The innermost circle shows the pangenome (green color), and the outer circles indicate the *V. parahaemolyticus* strains FORC_022 (orange), CDC_K4557 (red), and RIMD2210633 (dark brown).

VPaIs in FORC_022 and CDC_K4557 were identified by BLAST search against RIMD 2210633 strain, a clinical isolate used to identify genomic islands that could be markers for pandemic clones (**Table 2**) (Hurley et al., 2006). Four VPaIs were detected in the FORC_022 genome, as follows: VPaI-1 (FORC22_0350-FORC22_0352, FORC22_0358-FORC22_0360, FORC22_0362, FORC22_0365-FORC22_0368, and FORC22_0372-FORC22_0373), VPaI-3 (FORC22_1007-FORC22_1009 and FORC22_1034-FORC22_1041), VPaI-6 (FORC22_4366), and VPaI-7 (FORC22_4405 and FORC22_4406). VPaI-1 was reported to be unique to the pandemic group of *V. parahaemolyticus* strains isolated after 1995 (Hurley et al., 2006). VPaI-3 has been shown to encode integrase, signal transduction histidine kinase, helicase, methyl accepting chemotaxis protein, AcrBDF family protein as well as hypothetical proteins, and VPaI-6 encode putative virulence genes, such as hydrolases, cytotoxin integrase, and colicins. Broberg et al. (2011) also demonstrated that T3SS2 is encoded by the VPaI region, which is now referred to as VPaI-7. Our results showed that FORC_022 harbored T3SS and VPal-7 as well, accompanied by cytotoxicity in colon epithelial cells, implying that T3SS may be a major virulence factor in the FORC_022 strain. In addition, different virulence factors were detected in pangenome analysis of FORC_022, CDC_K4557, and RIMD22106333 (**Figure 3**). Three virulence factors of virulence-associated E, Type II/IV secretion systems, and Type IV pilin PilA were detected only in FORC_022. Therefore, VPaIs and additional virulence factors in FORC_022 may play important roles in the pathogenicity of this strain.

**Table 2:** Genomic islands (GIs) of *V. parahaemolyticus* FORC_022 and CDC_K4557, predicted by comparing the GIs in *V. parahaemolyticus* RIMD2210633.

ORFs of RIMD2210633	Annotation (chromosome)	FORC_022		CDC_K4557	
		Location	Detected gene number (Identity %)	Location	Detected gene number (Identity %)
**Genomic Islands (GIs)**					
VP0380–VP0403	VPaI^∗^-1 (Chromosome I)	367725–370261, 375474–378670, 380436–381926, 384740–390666, and 394100–395834 (FORC22_0350–FORC22_0352, FORC22_0358–FORC0360, FORC22_0362, FORC22_0365–FORC22_0368, and FORC22_0372–FORC22_0373)	VP0380–VP0382, VP0384–VP0386, VP0388, VP0395–VP0400, and VP0402–VP0403 (98%)	390403–391803, 402506–405945, and 412476–414155 (M636_19830, M636_19835, M636_19840, M636_19795, M636_19790, and M636_19880)	VP0380, VP0397–VP0400, and VP0402–VP0403 (94%)
VP0635–VP0643	VPaI-2 (chromosome I)	Absent		676971–678768, and 678888–685230 (M636_18615–M636_18640, and M636_18645–M636_18650)	VP0635–VP0643 (99%)
VP1071–VP1094	VPaI-3 (chromosome I)	1122265–1123693 and 1143337–1154849 (FORC22_1007-1009 and FORC22_1034-1041)	VP1073–VP1075, and VP1086–VP1094 (99%)	1161651–1161690, and 1161672–1170224 (M636_16310-M636_16335)	VP1088–VP1094 (99%)
VP2131–VP2144	VPaI-4 (chromosome I)	Absent		Absent	
VP2900–VP2910	VPaI-5 (chromosome I)	Absent		Absent	
VPA1253–VPA1270	VPaI-6 (chromosome II)	1437093–143761 (FORC22_4366)	VPA1253 (99%)	1373446–1373698 (M636_05645)	VPA1253 (99%)
VPA1312–VPA1398	VPaI-7 (chromosome II)	1472577–1473493 (FORC22_4405–FORC22_4406)	VPA1397–VPA1398 (99%)	1408874–1409790 (M636_05835–M636_05840)	VPA1397–VPA1398 (98%)

*^∗^Vibrio parahaemolyticus islands (VPaIs).*

### Differentially Expressed Genes in FORC_022 After Infection of Crabs

**FIGURE 4:**
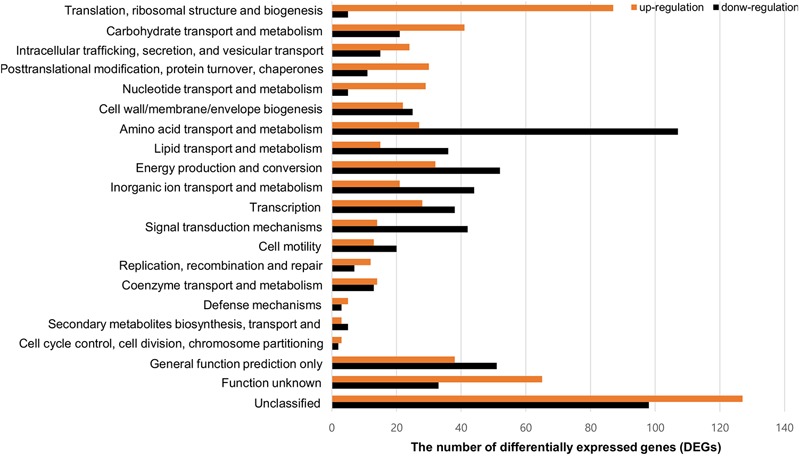
Functional analysis of differentially expressed gene (>2-fold change) in the FORC_022 strain exposed to crabs. Functional categories were determined by the Clusters of Orthologous Groups (COG) database. Genes upregulated (orange) and downregulated (black) after exposure to crabs are shown.

To compare gene expression in strains with or without contact with crabs, FORC_022 was exposed to washed swimming crabs in inoculation with minimal medium (mimic artificial seawater) and incubated for 4 h. The crabs tended to decompose after 6 h; thus, we analyzed samples after 4 h of incubation. Numerous genes were differentially expressed with significance in volcano plot (*p*-value < 0.01, two fold threshold; Supplementary Figure S7). A total of 1,283 genes were found to be differentially expressed between strains with or without contact with crabs (650 and 633 genes were upregulated and downregulated, respectively). Differently expressed genes were clustered into functionally related groups using the WebMGA server^5^ with the FORC_022 genome as the reference database (**Figure 4**). The top 15 overexpressed genes (over 5-fold change) from FORC_022 exposed to crabs are summarized in **Table 3**, and the top 15 down-regulated genes are presented in Supplementary Table S6.

**Table 3:** Top 15 up-regulated genes in the FORC_022 strain after exposure to crabs.

Locus tag	Product	Fold change	*p-*value^a^
FORC22_4640	Ornithine decarboxylase	89.96	0
FORC22_3982	Formate efflux transporter	81.91	0
FORC22_4639	Putrescine/proton symporter, putrescine/ornithine antiporter PotE	45.65	0
FORC22_4519	L-threonine 3-dehydrogenase	27.89	0
FORC22_4520	2-amino-3-ketobutyrate coenzyme A ligase	21.07	0
FORC22_2890	Aspartate ammonia-lyase	19.15	0
FORC22_3230	Multidrug resistance protein D	15.24	0
FORC22_2891	C4-dicarboxylate transporter DcuA	13.31	0
FORC22_3308	Outer membrane protein A precursor	11.44	0
FORC22_0045	Spermidine export protein MdtJ	10.22	0
FORC22_0046	Spermidine export protein MdtI	10.14	0
FORC22_1643	Type III secretion chaperone protein for YopD (SycD)	10.00	0
FORC22_4086	Formate dehydrogenase chain D	9.74	0
FORC22_2510	Outer membrane protein OmpU	9.24	0
FORC22_1642	Type III secretion host injection protein (YopB)	8.93	0

*^*a*^The p-value less than six decimal places were denoted as zero.*

The expression levels of genes related to amino acid transport and metabolism and lipid transport and metabolism were lower in the contact strain than in the strain not exposed to crabs, whereas the expression levels of genes related to carbohydrate transport and metabolism were higher in strains after contact with crabs. Genes related to biofilm formation such as tad locus, capsular polysaccharide (CPS), and lipopolysaccharide (LPS) (Yildiz and Visick, 2009), were overexpressed in the strain exposed to crabs. Therefore, FORC_022 could form biofilm to enhance its growth and survival in crabs, establishing a reservoir. To verify the overexpressed genes related to biofilm formation, we performed quantitative real-time reverse transcription polymerase chain reaction (qRT-PCR; Supplementary Figure S8). The amplified efficiencies for qRT-PCR were between 80.9 and 163.2%, and the efficiency curves were found to be linear with correlation coefficients (*R*^2^) ranging from 0.919 to 0.999. In addition, virulence factors, such as the type III secretion system, which exhibited cytotoxic activity toward human cells and was related to inflammatory diarrhea and septicemia (Caburlotto et al., 2010; Tamura et al., 2013), were also overexpressed in the strain following contact with crabs (**Table 3**).

**FIGURE 5:**
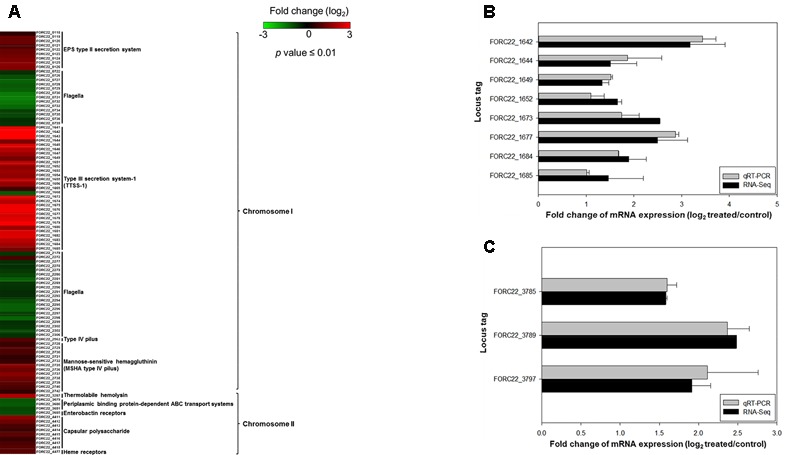
The relatively expression of virulence genes after exposure to crabs (*p*-value ≤ 0.01). **(A)** Fold changes of virulence genes in chromosomes I and II in heatmap. **(B)** Relative expression of the type III secretion system was determined by qRT-PCR. **(C)** Relative gene expression of the tight adhesion gene was determined. Error bars represent standard errors of the means from two independent experiments.

A comparison of expressed virulence genes between strains with and without contacted with crabs is presented in the form of a heatmap (*p*-value < 0.01; **Figure 5A**). This result showed that genes related to virulence factors, such as the EPS type II secretion system, type III secretion system, MSHA type IV pilus, thermolabile hemolysin (*tlh*), and heme receptors, were overexpressed in the contacted strain (Supplementary Table S7). The transcription levels of type III secretion system and tight adhesion genes were confirmed by real-time PCR using the same RNA extracts used for transcriptome analysis (**Figures 5B,C**). The levels detected by real-time PCR were consistent with the transcriptome results. These results suggested that the potential risk of foodborne illness by ingestion of contaminated crab with FORC_022 could be high due to its virulence factors.

## Conclusion

In the present study, we analyzed the complete genome sequences of *V. parahaemolyticus* FORC_022 strain isolated from soy sauce marinated crabs in South Korea. The genome of FORC_022 did not harbor *tdh* and *trh* genes, which are major virulence factors in clinically isolated *V. parahaemolyticus*. However, the cytotoxicity of FORC_022 was higher than the food poisoning causing strain (KCTC 2471), suggesting that other virulence factors may play a role in the pathogenesis of this infection. FORC_022 had additional virulence factors, such as accessory cholera enterotoxin, zona occludens toxin, and tad locus, compared with CDC_K4557 (the strain most closely related to FORC_022). In addition, the pandemic O3:K6 serotype specific gene (*toxRS*) and VPaI-1 were detected in FORC_022. Expression levels of adherence-, carbohydrate transporter-, and biofilm formation-related genes increased simultaneously after FORC_022 was exposed to crabs (**Table 3**). Therefore, this strain could survive and grow on crabs. In addition, several virulence factors, including the type III secretion system, tad locus, and thermolabile hemolysin, were overexpressed (**Figure 5**); this could lead to a higher risk of infection and pathogenicity (Morrison et al., 2012; Tamura et al., 2013). Although all of results in present study were analyzed by genomic data and gene expression, the genomic and transcriptomic results of FORC_022 provided new insights into our understanding of the molecular mechanisms mediating the survival and pathogenesis of *V. parahaemolyticus* in the crab products. Further studies including immunology analysis and animal model experiments are necessary to verify the pathogenicity of FORC_022 in present study.

## Author Contributions

K-HL, SR, HC, J-HL, HBK, HK, HJ, SC, and B-SK designed the study. HC, BL, and B-SK analyzed the sequencing data. HC, BL, and EN performed the experiments. HC, K-HL, SR, HY, J-HL, HBK, HK, HJ, SC and B-SK discussed the results and wrote the manuscript. All authors read and approved the final manuscript.

## Conflict of Interest Statement

The authors declare that the research was conducted in the absence of any commercial or financial relationships that could be construed as a potential conflict of interest.

## Footnotes

1 https://www.ncbi.nlm.nih.gov/genome/genomes/691

2 http://www.mgc.ac.cn/VFs/main.htm

3 https://server.gview.ca/

4 http://www.ncbi.nlm.nih.gov/

5 http://weizhong-lab.ucsd.edu/metagenomic-analysis/
