# Supplementary material for: Potential Survival and Pathogenesis of a Novel Strain, Vibrio parahaemolyticus FORC_022, Isolated From a Soy Sauce Marinated Crab by Genome and Transcriptome Analyses
Source: Front Microbiol. 2018 Jul 6;9:1504. doi: 10.3389/fmicb.2018.01504 (PMC6043650; doi:10.3389/fmicb.2018.01504)

***Supplementary Materials***

**Potential Survival and Pathogenesis of a Novel Strain, *Vibrio parahaemolyticus* FORC_022, Isolated from a Soy Sauce Marinated Crab by Genome and Transcriptome Analyses**

Han Young Chung^­1,2^, Byungho Lee^­1,2^, Eun Jung Na^1,2^, Kyu-Ho Lee^2,3^, Sangryeol Ryu^1,2^, Hyunjin Yoon^2,4^, Ju-Hoon Lee^2,5^, Hyeun Bum Kim^2,6^, Heebal Kim^2,7^, Hee Gon Jeong^2,8^, Bong-Soo Kim^2,9*^ and Sang Ho Choi^­1,2*^

*Correspondence:

Bong-Soo Kim

bkim79@hallym.ac.kr;

Sang Ho Choi

choish@snu.ac.kr

**Supplementary Table S1.** The verification of extracted RNA by Agilent 2100 Bioanalyzer

**Supplementary Table S2.** Oligonucleotides used for qRT-PCR in this study to verify transcriptome results

**Supplementary Table S3.** Amplification efficiency and correlation coefficient of qRT-PCR

**Supplementary Table S4.**Summary of *V. parahaemolyticus* FORC_022 genome

**Supplementary Table S5.** Detected virulence factors of *V. parahaemolyticus* FORC_022 against Virulence Factor database

**Supplementary Table S6.** Top 15 downregulated genes after the FORC_022 strain exposure to crab.

**Supplementary Table S7.** Transcriptional changes of virulence genes from FORC_022 after exposure to crab

**Supplementary Figure S1.** Transmission electron micrograph (TEM) image of *V. parahaemolyticus* FORC_022. The cells were negatively stained with 2.0% uranyl acetate (UA), for one minute. It was observed using TEM JEM-2100 (JEOL, Tokyo, Japan) at 200 kV.

**Supplementary Figure S2.** PCR assay for serotype specific genes in FORC_022 strain. (A) Results of amplification by O-serotypes specific genes (Chen et al., 2012). Lane 1: O1-serotype (target size: 474bp), 2: O2-serotype (238bp), 3: O3-serotype (868bp), 4: O4-serotype (671bp), 5: O5-serotype (852bp), 6: O6-serotype (1,409bp), 7: O7-serotype (385bp), 8: O8-serotype (680bp), 9: O9-serotype (419bp), 10: O10-serotype (343bp), 11: O11-serotype (524bp), and 12: O12-serotype (256bp). (B) Results of amplification by *toxRS* gene (target size: 1,470bp). M: 1kb plus DNA ladder.

**Supplementary Figure S3.** Genome tree of completely genome sequenced *V. parahaemolyticus* strains was obtained based on the average nucleotide identity (ANI) values. The ANI values were calculated using JSpecies, with nucleotide fragment length of 1,020 bp, based on BLAST algorithm. The tree was constructed using the R software. Scale bar estimated substitutions per site.

**Supplementary Figure S4.** Phylogenetic tree of FORC_022 with closely related *Vibrio* species was obtained based on 16S rRNA gene sequences. Phylogenetic tree was obtained by the neighbour-joining method with 1,000 bootstrap replicates. *Shewanella baltica* OS678 was used as an outgroup.The scale bar showed the number of nucleotide substitutions per site.

**Supplementary Figure S5.** Genome map of *V. parahaemolyticus* FORC_022. (A) Genome map of chromosome I. (B) Genome map of chromosome II. (C) Genome map of plasmid. The outer circle indicates the locations of all annotated ORFs, and the inner circle with the red peaks indicates GC content. Between these circles, the sky blue arrows indicate the rRNA operons, and the orange arrows indicate tRNAs. The GC skew (C-G)/(C+G) were calculated in an innermost circle (purple and green). All annotated ORFs are coloured differently according to the COG assignments. Genes with specialized functions are labeled with different colours as follows; virulence-related genes, red; prophage-related genes, blue; and other functional genes, black.

**Supplementary Figure S6.** Non-homologous regions between FORC_022 and CDC_K4557 strains. (A) Non-homologous region I included zona occludens toxin region, (B) Non-homologous region II included tight adhesion locus region.

**Supplementary Figure S7.** Volcano-plots of differentially expressed genes between strains with or without exposure to crabs. Number on the X- and Y-axis represent the fold change (log_2_) and *p* value (log_10_). Red dots represent differentially expressed genes.

**Supplementary Figure S8.** Relative levels of the biofilm related gene transcripts in the total RNA was determined by qRT-PCR. Error bars represent the SEM from two independent experiments.

**Supplementary Table S1.** The verification of extracted RNA by Agilent 2100 Bioanalyzer

|  | **Sample Name** | **Concentration (ng/ul)** | **Volume (ul)** | **Total amount (ug)** | **rRNA Ratio [23S/16S] [28S/18S]** | **Rin Value** |
| --- | --- | --- | --- | --- | --- | --- |
| 1^st^ set | Control strain^a^ | 496.5 | 22 | 10.9 | 1.9 | 8.5 |
|  | Treated strain^b^ | 518.3 | 22 | 11.4 | 1.8 | 8 |
| 2^nd^ set | Control strain | 605.6 | 22 | 13.3 | 1.6 | 9.9 |
|  | Treated strain | 501.7 | 22 | 11 | 1.7 | 8.8 |

^a^ FORC_022 strain without exposure to crab

^b^ FORC_022 strain with exposure to crab

**Supplementary Table S2.** Oligonucleotides used for qRT-PCR in this study to verify transcriptome results

| **Oligonucleotide** | **Sequence (5’→3’)^a^** | **Use** |
| --- | --- | --- |
| **For qRT-PCR** |  |  |
| FORC22_0204_F | GCAAACGGTGGCGGTTTATC | qRT-PCR of FORC22_0204 |
| FORC22_0204_R | TCACCACTTCCGAGATTGACTC |  |
| FORC22_0205_F | GTCGCTTTCTTAGGTCAACGAG | qRT-PCR of FORC22_0205 |
| FORC22_0205_R | CCCGCTCTCATGGCTTCTAA |  |
| FORC22_0208_F | TTCGCTTCCACAAGGACTACT | qRT-PCR of FORC22_0208 |
| FORC22_0208_R | CGGATAAATCGTTCGGCGAC |  |
| FORC22_0210_F | GAAAATGGTAATCCAATTCAGGTGC | qRT-PCR of FORC22_0210 |
| FORC22_0210_R | AAGACGCCCCAAGATAGAAAGT |  |
| FORC22_0211_F | CCCTGTTTTGCCGCTTTACTC | qRT-PCR of FORC22_0211 |
| FORC22_0211_R | CATCCCATAAGACAATCGCCCT |  |
| FORC22_0212_F | TTGCCAGGGCGAGAAGTATC | qRT-PCR of FORC22_0212 |
| FORC22_0212_R | ACACCGTCTACATGCCTAACTT |  |
| FORC22_1642_F | CGCTAAAAGAAGCGGGTGTG | qRT-PCR of FORC22_1642 |
| FORC22_1642_R | CAAAGGTCACGATTGCACCG |  |
| FORC22_1644_F | TGCTAACCGAAGCGAATTTTGT | qRT-PCR of FORC22_1644 |
| FORC22_1644_R | TGGTCCGCGTCATCTTTAGTT |  |
| FORC22_1649_F | CCATTGTGAACTGCTTCGTCC | qRT-PCR of FORC22_1649 |
| FORC22_1649_R | TTCCTGTGCGAGTTGCTGAA |  |
| FORC22_1652_F | GCCAATCCGGAAAGCAAACA | qRT-PCR of FORC22_1652 |
| FORC22_1652_R | AGTGTCGATCTGCGACCAAG |  |
| FORC22_1673_F | CGATCAGCGTGGTCAGAGAA | qRT-PCR of FORC22_1673 |
| FORC22_1673_R | CGTCAACCACTTGCTCAGGA |  |
| FORC22_1677_F | GTTGCTCGGAGATTTTGCTGG | qRT-PCR of FORC22_1677 |
| FORC22_1677_R | GCTTGTTCTTTCTGAGCGCC |  |
| FORC22_1684_F | TCTCGATTGGGCTTTCGCTT | qRT-PCR of FORC22_1684 |
| FORC22_1684_R | AACGCACTTTGGTCGGTTTG |  |
| FORC22_1685_F | ATGAAAACGCTGGAGGAGCA | qRT-PCR of FORC22_1685 |
| FORC22_1685_R | ATAGAGGACTTCGCCCCCTT |  |
| FORC22_3785_F | CAGGTTACAACATTTTTCCTGGTGA | qRT-PCR of FORC22_3785 |
| FORC22_3785_R | TTGTTAGAGACAAAACTGCGGC |  |
| FORC22_3789_F | GATGCCGAGGGTGCTCTTAG | qRT-PCR of FORC22_3789 |
| FORC22_3789_R | GCGTCTGGCTGTAGTTGAGA |  |
| FORC22_3797_F | GGCAGAAGAGGGGATTAGCG | qRT-PCR of FORC22_3797 |
| FORC22_3797_R | AAAAGCCCTGTCGATGCTGA |  |

^a^The oligonucleotides were designed using the *V. parahaemolyticus* FORC_022 genome sequence (GenBank^TM^ accession number CP13248, CP13249)

|  | Control | | Treated | |
| --- | --- | --- | --- | --- |
| Primer  (for qRT-PCR) | Amplification  Efficiency (%) | Correlation  Coefficient (R2) | Amplification  Efficiency (%) | Correlation  Coefficient (R2) |
| FORC22_0204 | 86.4 | 0.991 | 80.9 | 0.999 |
| FORC22_0205 | 90.5 | 0.986 | 97.8 | 0.999 |
| FORC22_0208 | 137 | 0.973 | 94.4 | 0.997 |
| FORC22_0210 | 94.8 | 0.995 | 93.2 | 0.998 |
| FORC22_0211 | 93.0 | 0.980 | 103.4 | 0.995 |
| FORC22_0212 | 89.4 | 0.983 | 101.8 | 0.994 |
| FORC22_1642 | 163.2 | 0.919 | 110.8 | 0.977 |
| FORC22_1644 | 125.8 | 0.951 | 98.6 | 0.996 |
| FORC22_1649 | 107.6 | 0.983 | 119.6 | 0.968 |
| FORC22_1652 | 112.1 | 0.943 | 126.3 | 0.973 |
| FORC22_1673 | 106.0 | 0.981 | 110.9 | 0.960 |
| FORC22_1677 | 123.5 | 0.941 | 98.3 | 0.996 |
| FORC22_1684 | 104.8 | 0.990 | 101.9 | 0.994 |
| FORC22_1685 | 115.3 | 0.928 | 116.2 | 0.992 |
| FORC22_3785 | 91.6 | 0.997 | 91.2 | 0.997 |
| FORC22_3789 | 102.1 | 0.998 | 100.1 | 0.996 |
| FORC22_3797 | 107.0 | 0.996 | 101.1 | 0.997 |
| Average | 108.8 | 0.973 | 102.7 | 0.990 |

**Supplementary Table S3.** Amplification efficiency and correlation coefficient of qRT-PCR

**Supplementary Table S4.**Summary of *V. parahaemolyticus* FORC_022 genome

| **Property** | **Term** |
| --- | --- |
| Finishing quality | Finished |
| Libraries used | Illumina 300 base pair paired-end library Roche 8 kb paired end library PacBio SMRTbell™ library (> 10 kb) for draft assembly |
| Sequencing platforms | Illumina MiSeq, 454 GS FLX Titanium, PacBio RS II |
| Assemblers | CLCbio CLC Genomics Workbench 7.5.1 Roche gsAssembler 2.6 PacBio SMRT Analysis 2.3.0 |
| Gene calling method | RAST ver. 2.0 (Glimmer 3), GeneMarkS |
| Average genome coverage | 326.62x |
| Chromosome length (bp) | 5,379,414 (PacBio and Illumina Miseq)  3,397,828 (Chromosome I)  1,879,989 (Chromosome II)  101,597 (Plasmid) |
| Contigs no. | 3 |
| Scaffolds no. | 3 |
| N50 | 3,397,828 |
| Locus Tag | FORC22 |
| Genbank ID | CP013248, CP013249, CP013250 |
| Genbank Date of Release | 2016-11-05 |
| BIOPROJECT | PRJNA301198 |
| Source Material Identifier | FORC_022 |
| Project relevance | Agricultural |

**Supplementary Table S5.** Detected virulence factors of *V. parahaemolyticus* FORC_022 against Virulence Factor database

| **Virulence factor** | **Annotation** | **Chromosome** | **Location** | **Function** |
| --- | --- | --- | --- | --- |
| **Adherence** |  |  |  |  |
| *mshH, mshI, mshJ, mshK, mshL, mshM, mshN, mshE, mshG, mshF, mshB, mshA, mshC, mshD, mshO mshP, mshQ* | Mannose-sensitive hemagglutinin (MSHA type IV pilus) | Chromosome I | 2953746-2966634 (FORC22_2728-FORC22_2744) | Hemagglutinin activity |
| *pilA, pilB, pilC, pilD* | Type IV pilus | Chromosome I | 2767326-2771646 (FORC22_2563-FORC22_2566) | Adhesion, motility |
| **Antiphagocytosis** |  |  |  |  |
| *cpsA, cpsB, cpsC, cpsD, cpsE, cpsF, cpsG, cpsH, cpsI, cpsJ* | Capsular polysaccharide | Chromosome II | 1479903-1491496 (FORC22_4411-FORC22_4421) | Protection |
| **Chemotaxis and motility** |  |  |  |  |
| *flaC, flaA, flgL, flgK, flgJ, flgI, flgH, flgG, flgF, flgE, flgD, flgC, flgB, flgA, flgM, flgN, flgP, flgO, flgT* | Flagella | Chromosome I | 800920-804968 (FORC22_0717-FORC22_0723) 807219-819746 (FORC22_0726-FORC22_0736) 820418-8215554 (FORC22_0739) | Flagella component |
| *flhB, fliR, fliQ, fliP, fliO, fliN, filM, fliL, fliK, fliJ, fliI, fliH, fliG, fliF, fliE, flrC, flrA, fliS, flaI, fliD, flaG, flaB, flaD, flaE, fliA, flhG, flhF, flhA* | Flagella | Chromosome I | 2446674-2477357 (FORC22_2279-FORC22_2307) | Flagella component |
| *cheR, cheV* | Chemotaxis protein | Chromosome I | 805141-806906 (FORC22_0723-FORC0724) | Chemotaxis |
| *cheW, cheB, cheA, cheZ, cheY* | Chemotaxis protein | Chromosome I | 2439615-2440109 (FORC22_2272) 2442023-2446640 (FORC22_2275-FORC22_2278) | Chemotaxis |
| *motA, motB, motY, motX* | Flagellar motor protein | Chromosome I | 717038-718762 (FORC22_0646-FORC22_0647) 2336730-2337611 (FORC22_2179) 3084307-3084942 (FORC22_2841) | Motility |
| **Iron uptake** |  |  |  |  |
| *vctA* | Enterobactin receptors | Chromosome II | 654353-656359 (FORC22_3685) | Ferric ion uptake |
| *irgA* | Enterobactin receptors | Chromosome I | 2848859-2850817 (FORC22_2639) | Ferric ion uptake |
| *hutA, hutR* | Heme receptors | Chromosome II | 1051751-1053832 (FORC22_4014) 1568680-1570818 (FORC22_4477) | Iron, heme uptake |
| *vctP, vctD, vctG, vctC* | Periplasmic binding protein-dependent ABC transport systems | Chromosome II | 649040-652607  (FORC22_3679-FORC22_3682) | Ferric ion uptake |
| **Quorum sensing** |  |  |  |  |
| *luxS* | Autoinducer-2 | Chromosome I | 2779697-2780215 (FORC22_2577) | Autoinducer production |
| *cqsA* | CAI-1 autoinducer synthase | Chromosome II | 724842-726023 (FORC22_3731) | Autoinducer production |
| **Secretion system** |  |  |  |  |
| *epsN, epsM, espL, epsK, epsJ, epsI, epsH, epsG, epsF, epsE, gspD, epsC* | EPS type II secretion system | Chromosome I | 134810-146130 (FORC22_0115-FORC22_0126) | Secretion system |
| *vopD, vopB, vcrH, vcrV, vcrG, vcrR, vcrD, vscY, vscX, sycN, tyeA, vopN, vscN, vscO, vscP, vscQ, vscR, vscS, vscT, vscU, vscL, vscK, vscJ, vscI, vscH, vscG, vscF, vscD, vscC, vscB, vscA, virF, virG, vxsC* | Type III secretion protein | Chromosome I | 1819885-1857460 (FORC22_1641-FORC22_1686) | Secretion system |
| *vopS, vopR, vopQ* | TTSS-1 secreted effectors | Chromosome I | 1840471-1841949 (FORC22_1665) 1842645-1843622 (FORC22_1668) 1844327-1845490 (FORC22_1671) | Secretion system |
| **Toxin** |  |  |  |  |
| *tlh* | Thermolabile hemolysin | Chromosome II | 229639-230895 (FORC22_3287) | pathogenesis |

**Supplementary Table S6.** Top 15 downregulated genes after the FORC_022 strain exposure to crab.

| **Locus tag** | **Product** | **Fold change** | ***p* value^a^** |
| --- | --- | --- | --- |
| FORC22_2794 | Acetylglutamate kinase | 0.0097 | 0 |
| FORC22_2795 | N-acetyl-gamma-glutamyl-phosphate reductase | 0.0106 | 0 |
| FORC22_2793 | Argininosuccinate synthase | 0.0177 | 0 |
| FORC22_1767 | Gamma-glutamyl-GABA hydrolase | 0.0183 | 0 |
| FORC22_2537 | Nitrogen regulatory protein P-II | 0.0194 | 0 |
| FORC22_1768 | Gamma-glutamyl-putrescine synthetase | 0.0227 | 0 |
| FORC22_3511 | Cobalt/zinc/cadmium efflux RND transporter, membrane fusion protein | 0.0237 | 0 |
| FORC22_2536 | Ammonium transporter | 0.0242 | 0 |
| FORC22_2028 | Trp operon leader peptide | 0.0275 | 0.00003 |
| FORC22_3512 | Heavy metal RND efflux outer membrane protein, CzcC family | 0.0298 | 0 |
| FORC22_0849 | TRAP-type C4-dicarboxylate transport system, periplasmic component | 0.0322 | 0 |
| FORC22_2384 | Sodium-dependent transporter | 0.0335 | 0 |
| FORC22_1764 | Gamma-glutamyl-putrescine oxidase | 0.0346 | 0 |
| FORC22_2689 | Ornithine carbamoyltransferase | 0.0354 | 0 |
| FORC22_1765 | Gamma-glutamyl-aminobutyraldehyde dehydrogenase | 0.0366 | 0 |

^a^The *p* value less than six decimal places were denoted as zero.

**Supplementary Table S7.** Transcriptional changes of virulence genes from FORC_022 after exposure to crab

| **Locus tag** | **Product** | **Fold change** | ***p* value^a^** |
| --- | --- | --- | --- |
| **Type III secretion system** | |  |  |
| FORC22_1641 | Type III secretion host injection and negative regulator protein (YopD) | 7.19 | 0 |
| FORC22_1642 | Type III secretion host injection protein (YopB) | 8.93 | 0 |
| FORC22_1643 | Type III secretion chaperone protein for YopD (SycD) | 10.00 | 0 |
| FORC22_1644 | type III secretion cytoplasmic LcrG inhibitor LcrV | 2.77 | 0 |
| FORC22_1645 | Type III secretion cytoplasmic plug protein LcrG | 5.86 | 0.00071 |
| FORC22_1646 | Type III secretion low calcium response protein LcrR | 4.28 | 0.00001 |
| FORC22_1647 | Type III secretion inner membrane channel protein | 3.42 | 0 |
| FORC22_1649 | Type III secretion protein SctX | 2.53 | 0.00660 |
| FORC22_1651 | Type III secretion outermembrane negative regulator of secretion TyeA | 4.63 | 0.00006 |
| FORC22_1652 | Type III secretion outermembrane contact sensing protein | 7.19 | 0.00001 |
| FORC22_1673 | Type III secretion cytoplasmic protein YscL | 5.82 | 0 |
| FORC22_1674 | Type III secretion cytoplasmic protein YscK | 4.42 | 0 |
| FORC22_1675 | Type III secretion bridge between inner and outermembrane lipoprotein | 6.95 | 0 |
| FORC22_1676 | Type III secretion cytoplasmic protein YscI | 7.18 | 0 |
| FORC22_1677 | Type III secretion effector protein YopR, | 5.70 | 0 |
| FORC22_1678 | Type III secretion spans bacterial envelope protein YscG | 6.03 | 0 |
| FORC22_1679 | Type III secretion cytoplasmic protein YscF | 7.67 | 0 |
| FORC22_1680 | Type III secretion protein YscE | 3.60 | 0.00055 |
| FORC22_1681 | Type III secretion inner membrane protein | 5.88 | 0 |
| FORC22_1682 | Type III secretion outermembrane pore forming protein | 6.06 | 0 |
| FORC22_1683 | Type III secretion chaperone protein for YopN (SycN,YscB) | 4.48 | 0 |
| FORC22_1684 | Type III secretion negative regulator LscZ | 3.69 | 0 |
| FORC22_1685 | type III secretion regulator ExsA | 2.59 | 0 |
| **Thermolabile hemolysin (TLH)** | |  |  |
| FORC22_3287 | Thermolabile hemolysin precursor | 3.99 | 0 |
| **Tight adhesion locus (Tad locus)** | |  |  |
| FORC22_3784 | Flp pilus assembly protein, pilin Flp | 3.90 | 0 |
| FORC22_3785 | Type IV prepilin peptidase TadV/CpaA | 3.00 | 0 |
| FORC22_3786 | Flp pilus assembly protein RcpC/CpaB | 3.34 | 0 |
| FORC22_3787 | Type II/IV secretion system secretin RcpA/CpaC | 4.10 | 0 |
| FORC22_3788 | hypothetical protein | 5.01 | 0 |
| FORC22_3789 | Type II/IV secretion system ATPase TadZ/CpaE, | 5.60 | 0 |
| FORC22_3790 | Type II/IV secretion system ATP hydrolase TadA/VirB11/CpaF | 6.52 | 0 |
| FORC22_3791 | Flp pilus assembly protein TadB | 7.34 | 0 |
| FORC22_3792 | Type II/IV secretion system protein TadC | 7.58 | 0 |
| FORC22_3793 | Flp pilus assembly protein TadD, contains TPR repeat | 6.51 | 0 |
| FORC22_3794 | Flp pilus assembly membrane protein TadE | 3.96 | 0 |
| FORC22_3795 | Flp pilus assembly surface protein TadF, ATP/GTP-binding motif | 2.08 | 0 |
| FORC22_3796 | Protein TadG, associated with Flp pilus assembly | 4.00 | 0 |
| FORC22_3797 | Outer membrane lipoprotein precursor, OmpA family | 3.81 | 0 |

^a^The *p* value less than six decimal places were denoted as zero.

**Supplementary Figure S1.** Transmission electron micrograph (TEM) image of *V. parahaemolyticus* FORC_022. The cells were negatively stained with 2.0% uranyl acetate (UA), for one minute. It was observed using TEM JEM-2100 (JEOL, Tokyo, Japan) at 200 kV.


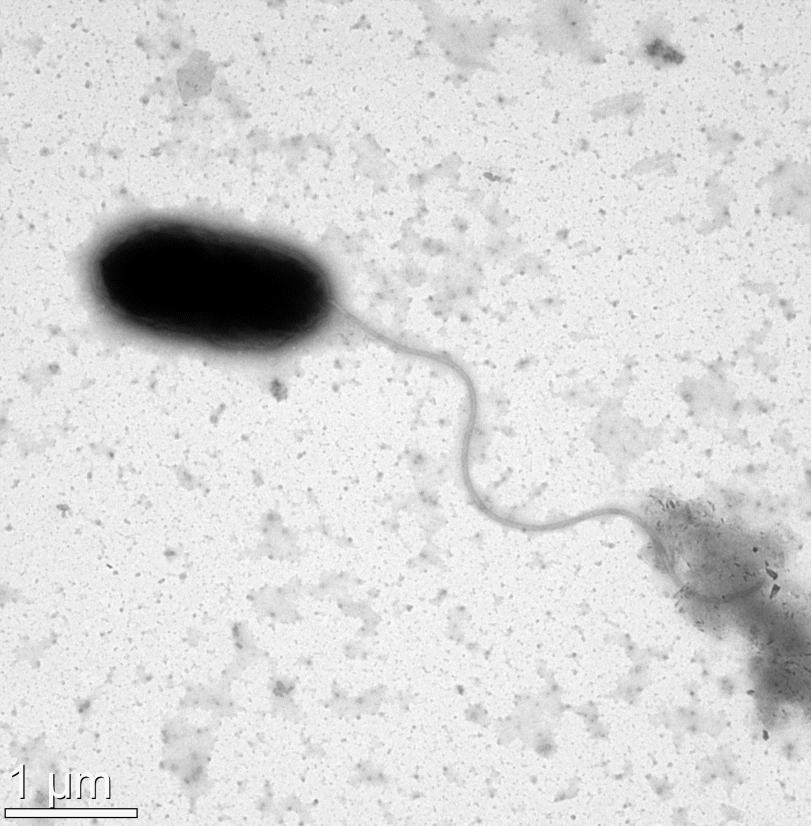


**Supplementary Figure S2.** PCR assay for serotype specific genes in FORC_022 strain. (A) Results of amplification by O-serotypes specific genes (Chen et al., 2012). Lane 1: O1-serotype (target size: 474bp), 2: O2-serotype (238bp), 3: O3-serotype (868bp), 4: O4-serotype (671bp), 5: O5-serotype (852bp), 6: O6-serotype (1,409bp), 7: O7-serotype (385bp), 8: O8-serotype (680bp), 9: O9-serotype (419bp), 10: O10-serotype (343bp), 11: O11-serotype (524bp), and 12: O12-serotype (256bp). (B) Results of amplification by *toxRS* gene (target size: 1,470bp). M: 1kb plus DNA ladder.


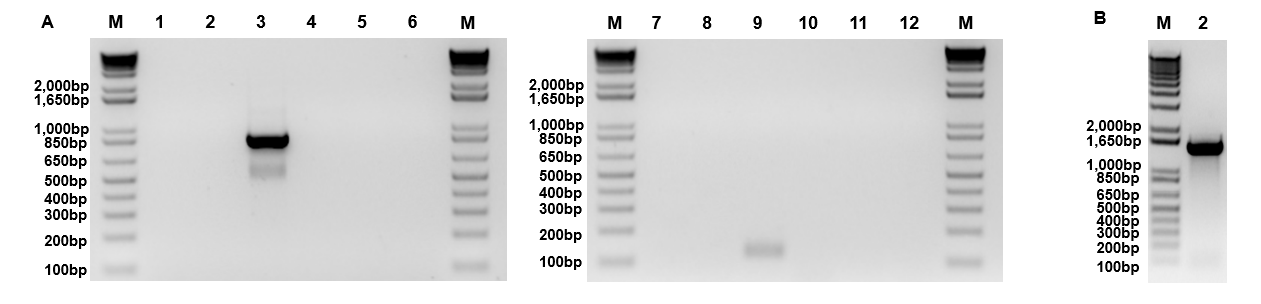


**Supplementary Figure S3.** Genome tree of completely genome sequenced *V. parahaemolyticus* strains was obtained based on the average nucleotide identity (ANI) values. The ANI values were calculated using JSpecies, with nucleotide fragment length of 1,020 bp, based on BLAST algorithm. The tree was constructed using the R software. Scale bar estimated substitutions per site.


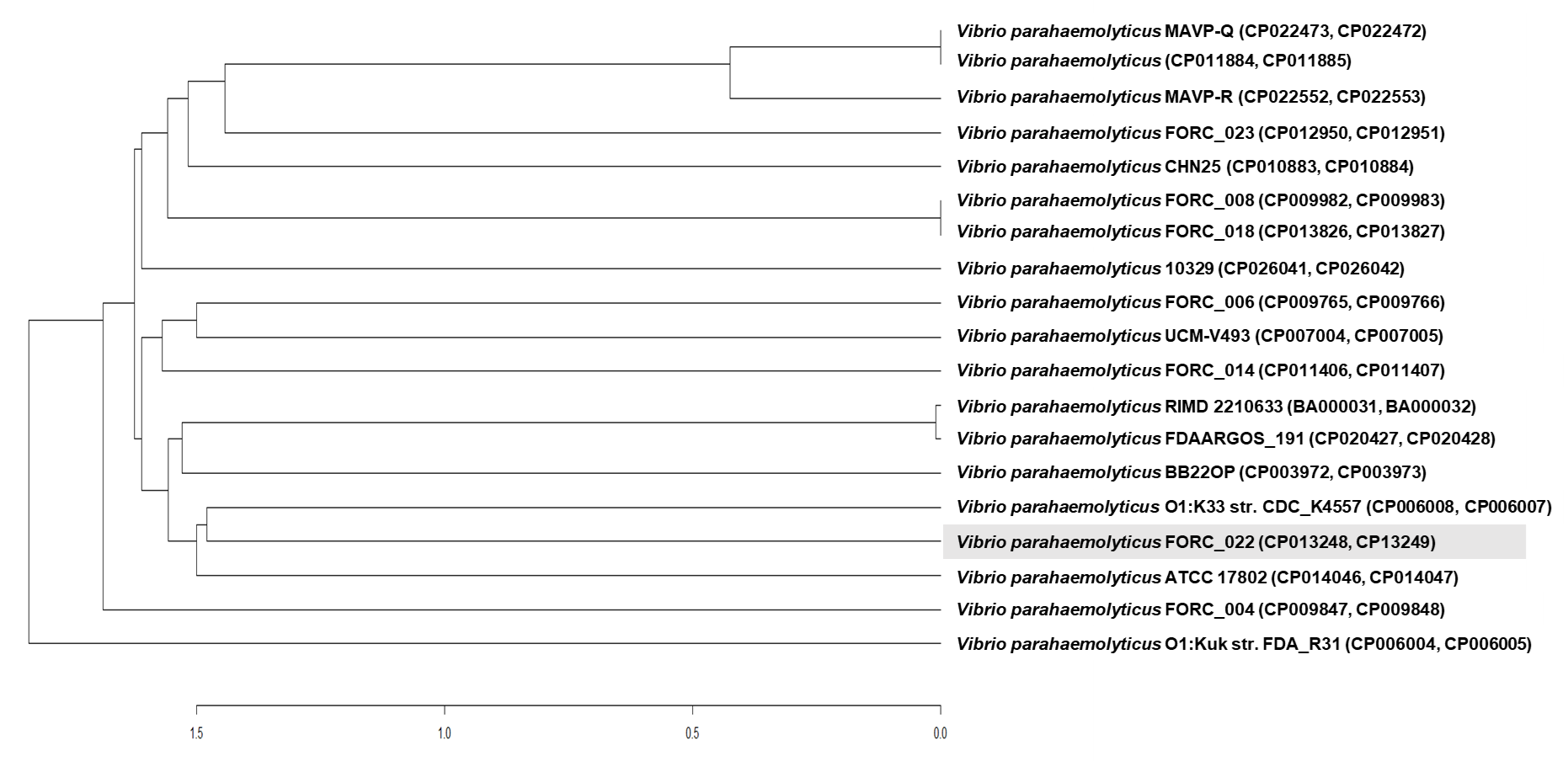


**Supplementary Figure S4.** Phylogenetic tree of FORC_022 with closely related *Vibrio* species was obtained based on 16S rRNA gene sequences. Phylogenetic tree was obtained by the neighbour-joining method with 1,000 bootstrap replicates. *Shewanella baltica* OS678 was used as an outgroup.The scale bar showed the number of nucleotide substitutions per site.


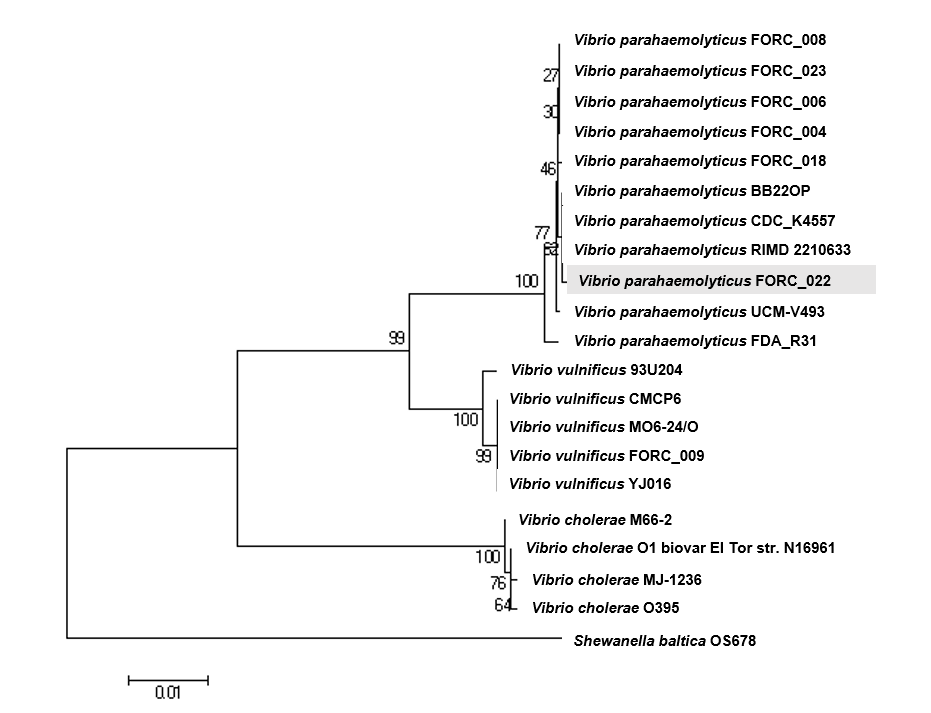


**Supplementary Figure S5.** Genome map of *V. parahaemolyticus* FORC_022. (A) Genome map of chromosome I. (B) Genome map of chromosome II. (C) Genome map of plasmid. The outer circle indicates the locations of all annotated ORFs, and the inner circle with the red peaks indicates GC content. Between these circles, the sky blue arrows indicate the rRNA operons, and the orange arrows indicate tRNAs. The GC skew (C-G)/(C+G) were calculated in an innermost circle (purple and green). All annotated ORFs are coloured differently according to the COG assignments. Genes with specialized functions are labeled with different colours as follows; virulence-related genes, red; prophage-related genes, blue; and other functional genes, black.


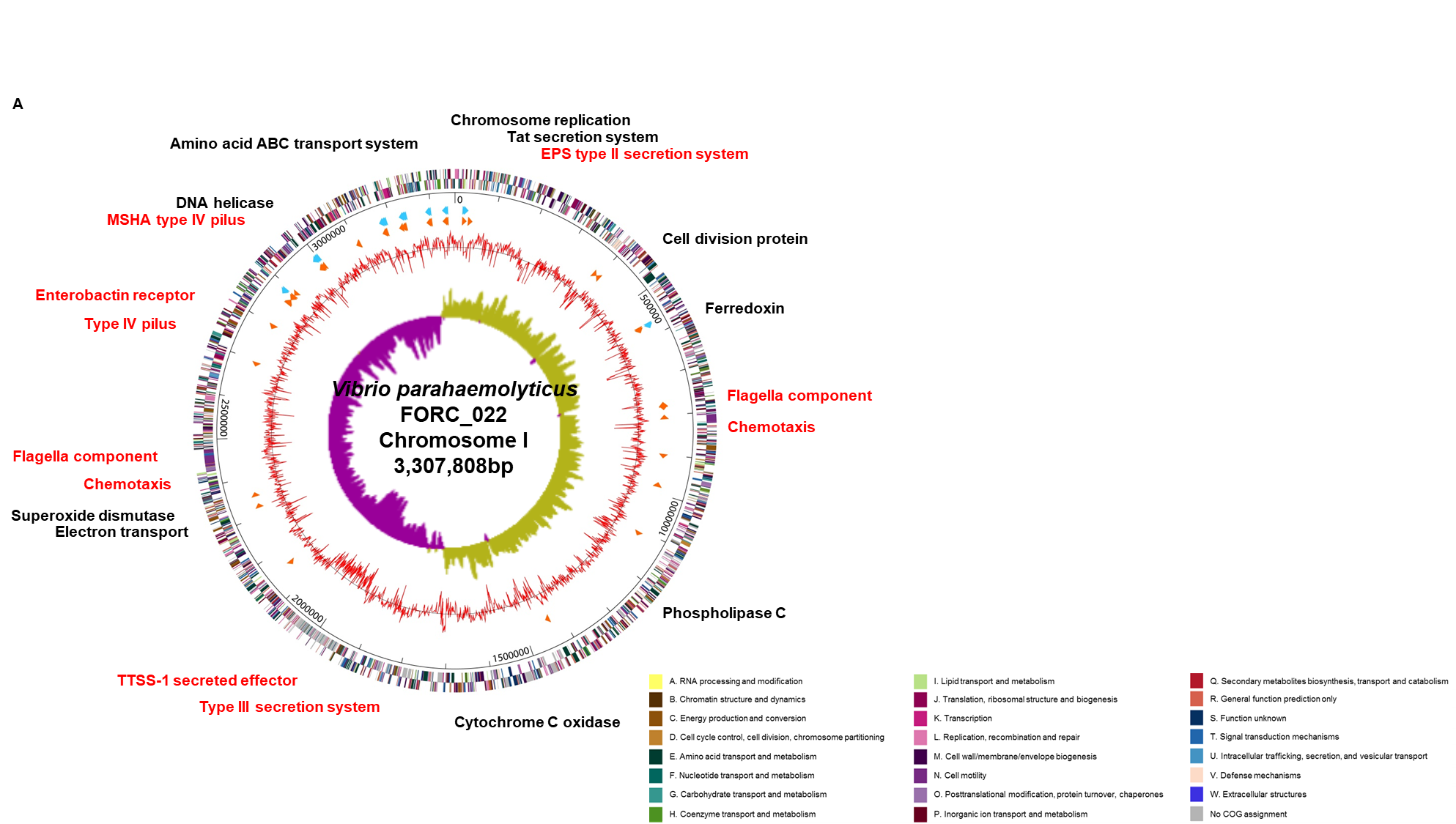

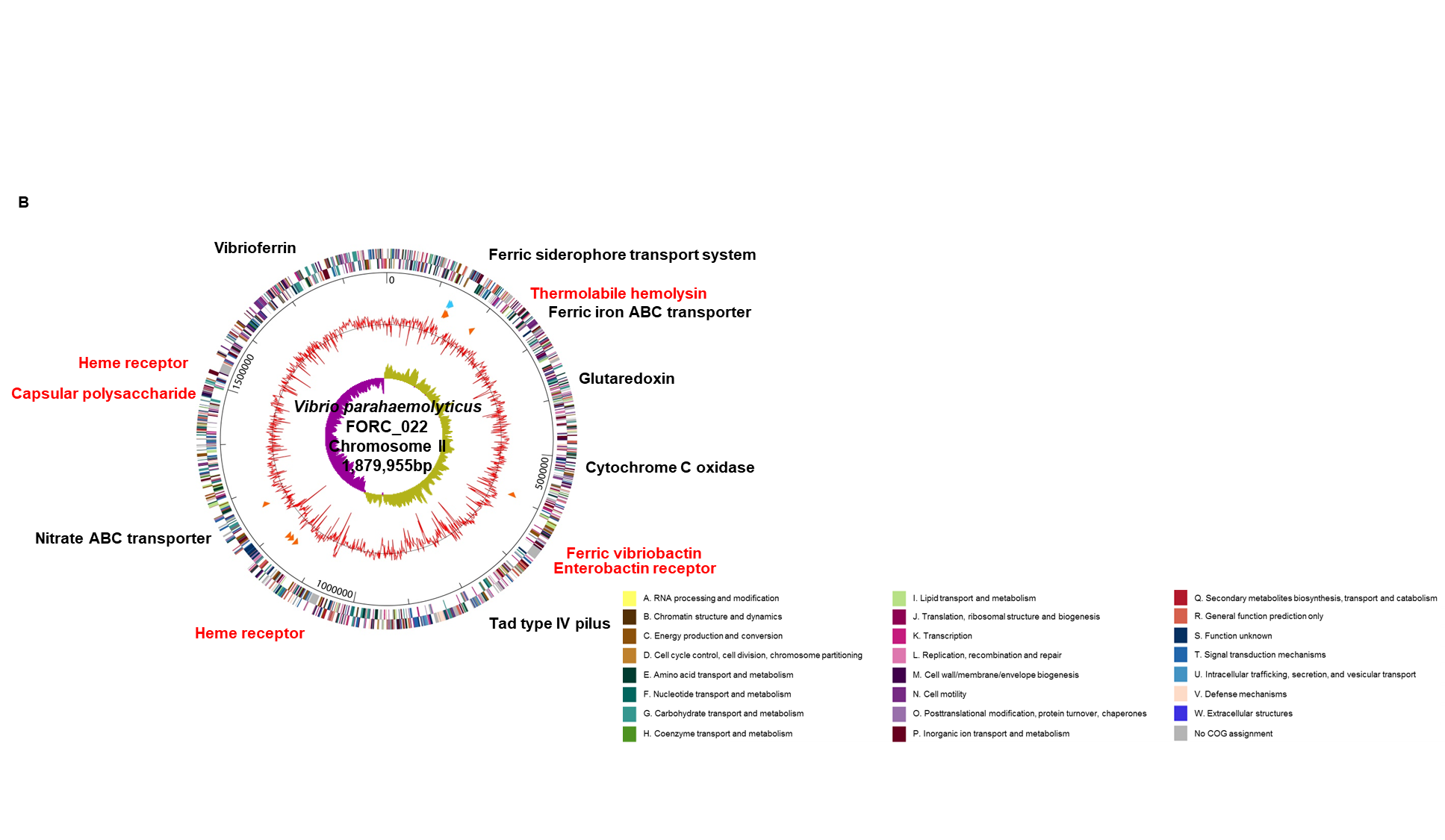

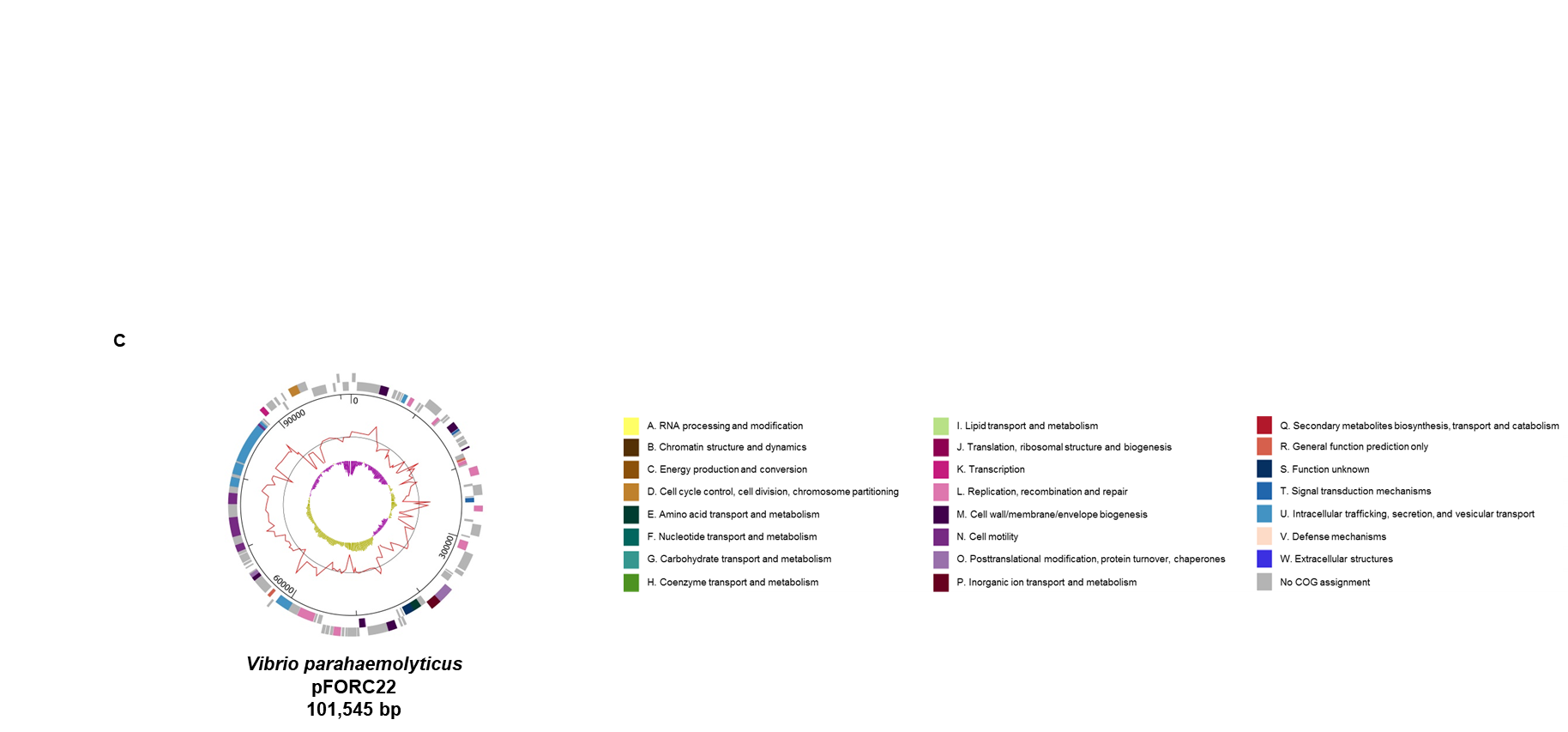


**Supplementary Figure S6.** Non-homologous regions between FORC_022 and CDC_K4557 strains. (A) Non-homologous region I included zona occludens toxin region, (B) Non-homologous region II included tight adhesion locus region.


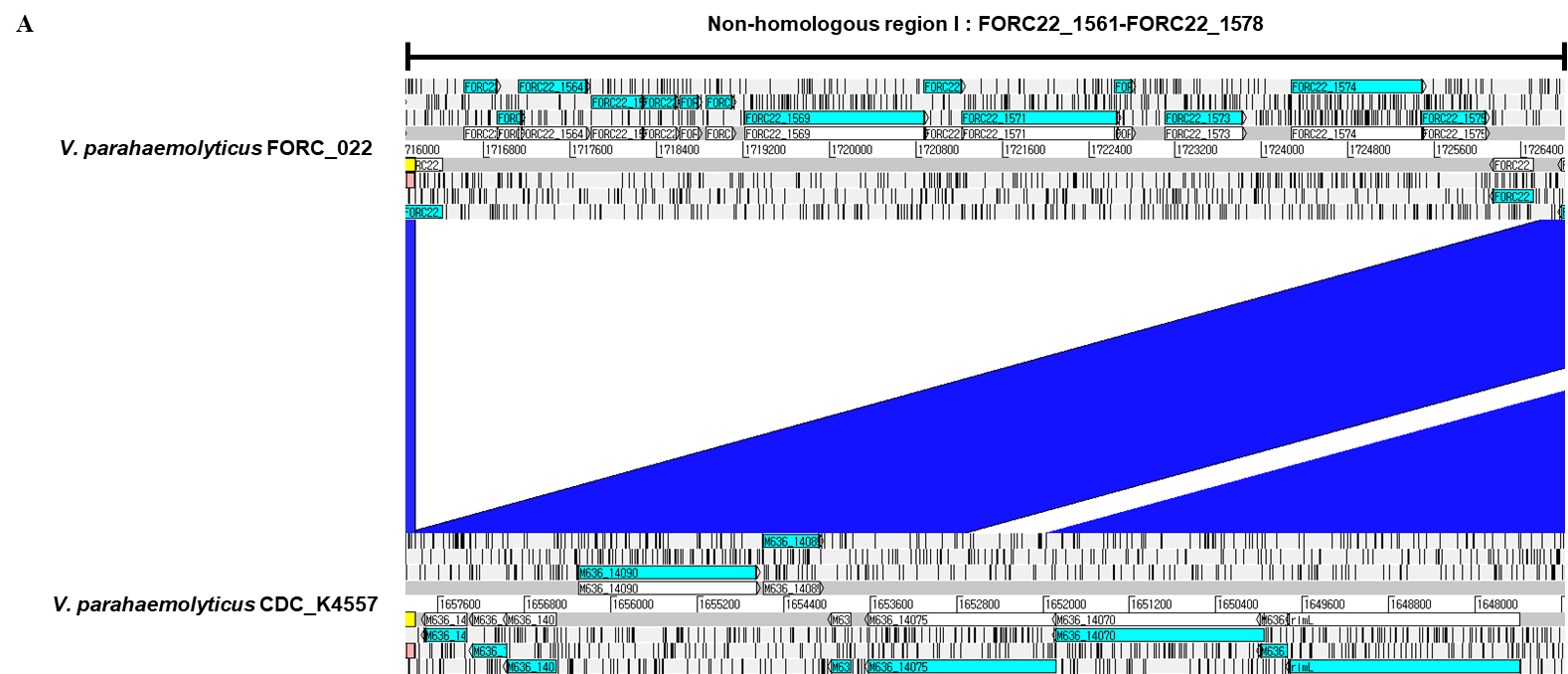

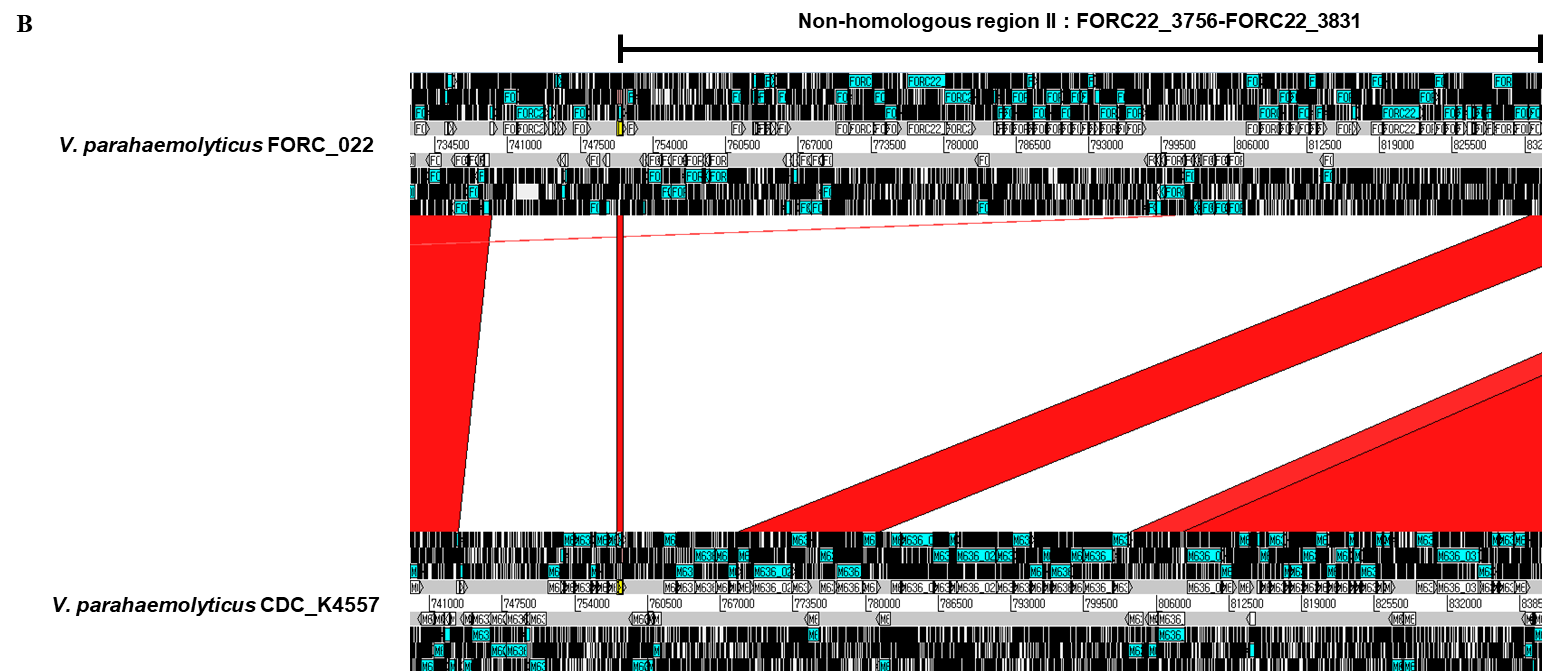


**Supplementary Figure S7.** Volcano-plots of differentially expressed genes between strains with or without exposure to crabs. Number on the X- and Y-axis represent the fold change (log_2_) and *p* value (log_10_). Red dots represent differentially expressed genes.


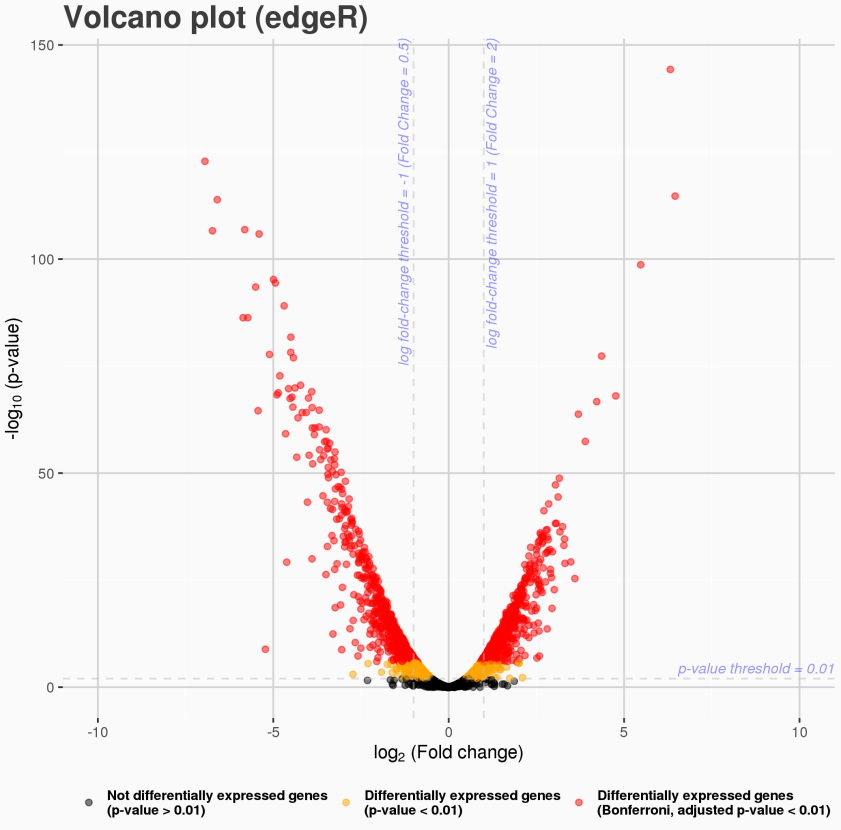


**Supplementary Figure S8.** Relative levels of the biofilm related gene transcripts in the total RNA was determined by qRT-PCR. Error bars represent the SEM from two independent experiments.


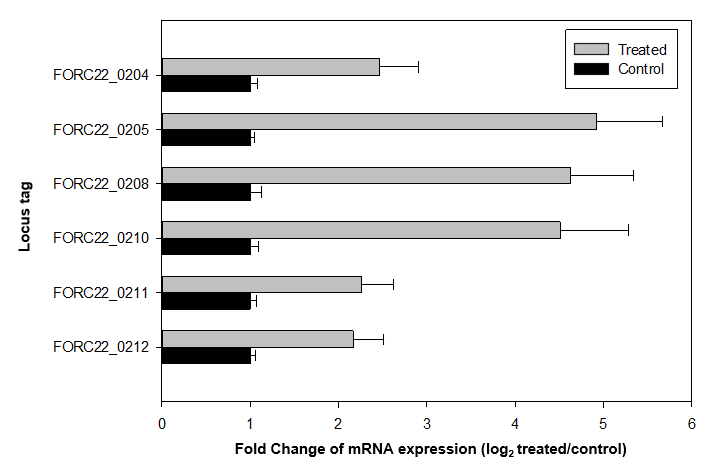

Supplement: Supplementary file 1 [file Data_Sheet_1.docx]
